# Overcoming challenges to reduce time to antibiotic therapy in febrile neutropenic children: insights from a Mexican center

**DOI:** 10.1016/j.htct.2024.04.123

**Published:** 2024-08-18

**Authors:** Julia Esther Colunga-Pedraza, Ingrid Gabriela Lopez-Reyna, Denisse Natalie Vaquera-Aparicio, Samantha Paulina Peña-Lozano, Jafet Arrieta, Lucía Elizabeth Hernández-Torres, Perla Rocío Colunga-Pedraza, Mónica Regalado, Yajaira Valentine Jiménez-Antolinez, Fernando García-Rodríguez, Oscar González-Llano

**Affiliations:** a Department of Hematology. Monterrey, Universidad Autónoma de Nuevo León, Hospital Universitario ¨Dr. José Eleuterio González¨, México; b Institute for Healthcare Improvement, Boston, USA

**Keywords:** Febrile neutropenia, Golden hour, Hematology–oncology, Pediatrics, Low-middle-income country

## Abstract

**Background:**

Providing quality supportive therapy for children with cancer is essential to reduce the high mortality rates in low- and middle-income countries. Febrile neutropenia is the most common life-threatening complication of cancer in children. The objective of this study was to evaluate the long-term effectiveness of the ‘Golden Hour’ intervention in reducing the time to administer antibiotics and its impact on clinical outcomes in a Mexican hospital.

**Methods:**

A comparative study of children with febrile neutropenia who attended the emergency department at the Hospital Universitario “Dr. José Eleuterio González” was performed between January 2017 and December 2022. In May 2019, this center joined the collaborative ‘Mexico in Alliance with St. Jude’ project. An adapted improvement program was developed based on the implementation of an algorithm comprising institutional guidance, supplies kit, standardization of sample processing, training of healthcare providers, and patient education. The time to antibiotic administration was compared with clinical outcomes between the historical control and post-intervention groups.

**Results:**

A total of 291 patients were included, 122 in the pre-intervention period and 169 in the intervention period. Only 5.7 % of the pre-intervention group received the first dose of antibiotics within 60 min of presenting to the emergency department compared to 84.6 % in the intervention group (*p*-value <0.000). The median times to antibiotic administration in the pre-intervention and post-intervention periods were 269.4 and 50.54 min, respectively (*p*-value <0.000). Clinical deterioration and admission to the pediatric intensive care unit decreased significantly from 6.6 % to 2.3 % (*p*-value = 0.03).

**Conclusions:**

Sustainability of the quality improvement project ‘Golden Hour’ in low- to mid-income countries demonstrated high effectiveness in reducing time to antibiotic administration among children with febrile neutropenia and improved clinical outcomes over three years of implementation.

## Introduction

Cancer is the main cause of death in children and adolescents.^1^ According to the World Health Organization (WHO), approximately 400,000 new cases are diagnosed annually worldwide; about 8 in 10 of these children live in low- and middle-income countries (LMICs).^2^ In Mexico, approximately 5000 new cases are diagnosed annually.^3^ According to the National Institute of Statistics and Geography (INEGI), between 2011 and 2016, 50 % of cancer mortality among 0–17-year-old patients was associated with leukemia.^4^

In high-income countries (HICs), the five-year cancer survival rate in children is reported to be up to 85 %, whereas in LIMCs the survival rate is below 50 %. ^5^ The main reasons contributing to this gap include delayed diagnosis, treatment abandonment, and treatment-related complications with the main chemotherapy-associated complication being febrile neutropenia (FN).^6^ Infectious complications remain major contributors to adverse outcomes in limited settings.^7^

Recognizing this global concern and aiming to bridge the gap in childhood cancer survival rates between LMICs and developed countries, strategic alliances have been formed at a global level. One such alliance is the Mexico in Alliance with St. Jude (MAS) project, which is a multi-site, intersectoral collaboration. The primary objective of this partnership is to establish early and optimal management of complications, thereby reducing mortality associated with treatment toxicity. Ultimately, this initiative aims to improve the survival and quality of life for children and adolescents with cancer worldwide.^8^

One-third of pediatric patients undergoing chemotherapy develop FN.^9^ Torres et al.^10^ reported a 27 % rate of treatment-related mortality associated with infectious complications at the Instituto Nacional de Cancerologia (INCan) in Mexico, whereas Silva et al.^11^ reported a rate of 17 % in Brazil,^12^ and in Guatemala a 24 % mortality rate has been reported. On the other hand, in HICs, mortality due to FN events with adequate treatment is 2 %.^13^, ^14^, ^15^

Although fever can have multiple etiologies, an infection is identified in 48–60 % of cases with up to 20 % of these patients developing bacteremia.^16^ Therefore, fever in pediatric hematology-oncology patients (PHOPs) is considered an oncological emergency.^9^ According to the guidelines of the American Society of Infectious Diseases (IDSA), intravenous antibiotics should be administered within the shortest possible time from the onset of fever to reduce infection-related morbidity and mortality.^17^, ^18^, ^19^, ^20^ The ideal time for antibiotic administration has not been well-defined, especially in PHOPs, however, some authors recommend less than 60 min.^20^ In adult patients with septic shock, administration of antibiotics within the first 60 min of documented hypotension improves prognosis, and late administration is associated with a 7.6 % decrease in the survival rate for each hour of delay.^21^ Moreover, late antibiotic therapy leads to prolonged hospitalization to manage complications and, therefore, higher costs.^22^ For this, oncological centers, especially in LMICs should prioritize strategies to promote the timely detection of fever and the administration of antibiotics in PHOPs with FN thereby contributing to reduce mortality and costs.

Implementing an educational program in LMICs comes with many challenges. In Mexico there is very little training in quality improvement; caregivers have low levels of education and the socioeconomic status of the population is low.

Our center is a tertiary care hospital with 1000 beds providing care to adult and pediatric patients, including maternity services, so there are many medical emergencies and a shortage of health care staff. The Pediatric Emergency Department comprises two inpatient floors with 20 beds each and receives patients from all specialties. As a university hospital, a significant proportion of our medical and nursing staff are in training, leading to extensive personnel rotation.

## Objective

This study aimed to evaluate the effectiveness of the ‘Golden Hour’, a quality improvement project implemented in the context of MAS collaborative project. The goal of the ‘Golden Hour’ is to reduce the time to antibiotic therapy (TTA) to up to 60 min among PHOPs with fever who present to the emergency department (ED) and assess the impact on outcomes.

## Methods

Our hospital actively participated in the 1st MAS Collaborative Project (May 2019–November 2020) and is currently participating in the 2nd (November 2021–May 2023). This project, following the breakthrough series model of the Institute for Healthcare Improvement, aims to increase the percentage of PHOPs with FN who receive the first antibiotic dose within 60 min of presenting to the ED. This model involves a multidisciplinary team working toward a shared goal and a common theory of change with measurement strategy; it is organized around learning sessions and action periods. During action periods, teams test changes in ideas using Plan-Do-Study-Act (PDSA) cycles based on theory of change; attend monthly learning calls; report Plan-Do-Study-Acts and data in a shared repository; and receive continuous support and coaching.^23^

### Study design

This observational study included PHOPs receiving treatment at Hospital Universitario “Dr. Jose Eleuterio Gonzalez” of the Universidad Autónoma de Nuevo León who presented to the ED with FN between January 2017 and December 2022. Group A involved patients who presented to the ED during the pre-intervention period (January 2017–May 2019) with data being collected retrospectively. Alternatively, Group B comprised patients who presented to the ED during the intervention period (May 2019–2022) with data being collected prospectively from collaborative records; the clinical follow-up of the patient was conducted at 24, 48, and 72 h after admittance, as well as at discharge.

### Definition

FN was considered when a PHOP had a axillar temperature >38.5 °C in one measurement or >38 °C on two or more occasions within 12 h, and a total neutrophil count ≤500 cells/µL or from 500 to 1000 cells/µL with a decrease expected within the following 48 h.^24^

According to the clinical practice guidelines for the use of antimicrobial agents in neutropenic patients with cancer by the IDSA published in 2010, the risk of patients should be assessed at the time of fever presentation. High-risk Patients, that is, patients with prolonged neutropenia lasting >7 days or profound neutropenia with an absolute neutrophil count ≤100 cells/µL after cytotoxic chemotherapy should be admitted to a hospital unit, as should those with significant comorbidities such as hypotension, pneumonia, acute abdominal pain, or neurological alterations. Patients at low risk include those with short neutropenia expected to last ≤7 days, with no or few comorbidities, and who are candidates for oral empirical therapy.^25^ In our center, low-risk patients are treated with oral levofloxacin and re-evaluated every 24 h in the outpatient clinic. These patients were not included in this study.

### Quality improvement measures

TTA - Time between triage and administration of the first dose of antibiotic;.

TTI - Time between triage and indication of the first dose of antibiotic.

TIA - Time between indication and administration of the first dose of antibiotic.

The date and time of triage are automatically obtained and recorded in the electronic medical record, as are the dates and times of antibiotic prescription by the physician, preparation, and administration.

### Measures of clinical effectiveness

Sepsis: Percentage of patients developing sepsis within the first 48 h from the initial clinical assessment.

Critical Interventions: Percentage of patients receiving critical-level interventions (requirement for aminergic and/or ventilatory support) within the first 48 h from the initial clinical assessment.

Transfer to intensive care unit (ICU): Percentage of patients transferred to the ICU within the first 48 h from the initial clinical assessment.

Mortality of patients: 30-Day mortality rate.

### Improvement strategies implemented

#### Warning data and temperature taking

All patients recently diagnosed with childhood cancer and those starting intensive phases of chemotherapy and their parents received an introductory talk on warning data related to the condition of the PHOP, highlighting the importance of going immediately to hospital in case of fever and performing a preventive action plan. A kit with a thermometer was provided with written instructions and parents and patients were taught to record the temperature.

#### Identification of the patient at risk

This program starts with patient consultation to identify high-risk patients (neutrophils <500 cells/µL at the time of the consultation or patients for whom, according to their chemotherapy cycle, profound neutropenia can be expected during the following ten days). These patients were given a red card with their data, treatment phase, last neutrophil count at consultation, somatometry, and recommendations for parents.

This red card facilitates the identification of patients when they arrive for emergency treatment, granting them a direct pass to the pediatric ED and the start of the ‘golden hour’ protocol.

#### Training

Every six months staff, including undergraduate medical interns, residents, specialists, nursing staff, and interns, who join the ‘golden hour’ team, received training. These training sessions were scheduled in a pediatric service auditorium.

#### The arrival of the febrile patient at the emergency department

**Figure 1:**
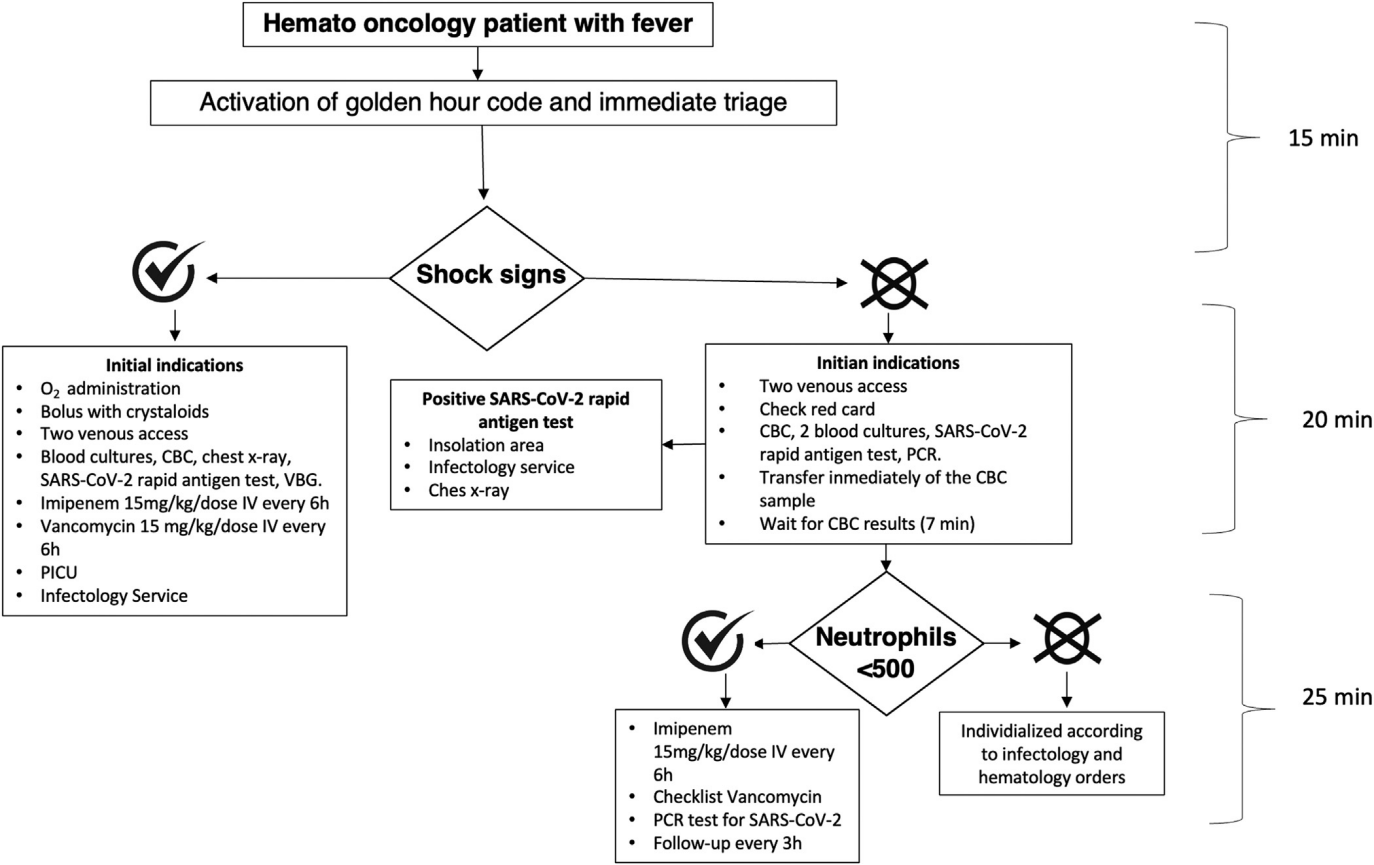
Golden Hour local algorithm. * CBC: Complete blood count; SARS-CoV-2: Severe acute respiratory syndrome coronavirus 2; VBG: Venous blood gas; PICU: Pediatric intensive care unit; PCR: Polymerase chain reaction.

The staff of the ED, including doctors, nurses, social workers, and guards, as well as parents were informed about the protocol. When a patient with these characteristics arrived, the ‘golden code’ was activated. The mother was interviewed about the onset of fever and data were collected from the patient's file. The complete algorithm is illustrated in Figure 1.

The antibiotics used before the ‘Golden Hour’ program were chosen based on patient characteristics. Antibiotic selection was made after consultation with the infectious diseases service based on local resistance studies; hence, it was determined that all patients with FN could receive imipenem initially and subsequently and according to the clinical evolution of the patient, the bacterial isolation, and the antibiogram, some modifications would be made to the antibiotic scheme.

#### Venous access

Simultaneously, a program was implemented to increase the percentage of children with port catheters with staff training sessions being conducted to improve access.

#### Golden kit

A care kit was organized and frequently replenished with the necessary supplies for the treatment of three fever and neutropenia events.

#### Blood cultures collection standardization

Systematically, one peripheral and one central blood sample were drawn for cultures in the post-intervention group before the initiation of antibiotics. It was also ensured that the golden kit had culture media available at all times.

#### Laboratory processing

In conjunction with laboratory personnel, the processing of hematic biometry was prioritized aiming to report the neutrophil count in less than ten minutes.

These strategies, tested in parallel with other members of the MAS collaborative team, demonstrated a high level of reliability that has been sustained over the three years of the project.

#### Statistical analysis

Statistical analysis was performed using the Statistical Package for Social Sciences (SPSS) Statistics software version 26. Comparative tests were carried out to study the difference in clinical outcomes between Groups A and B. Nominal dichotomous variables were compared using Fisher's exact test (proportion of patients receiving antibiotic therapy in ≤60 min, proportion of patients who developed sepsis or required intensive therapy, vital status).

## Results

A total of 291 patients were included in this study. Male sex predominated at 60.5 % (*n* = 176). The median age of the patients was five years (range: 1–15 years). The main diagnoses reported were acute lymphoblastic leukemia (57.7 %), solid tumors (19.6 %) and acute myeloid leukemia (AML) in 9.3 %.

Patients were classified in two cohorts; Group A consisted of 122 patients seen before the implementation of the ‘Golden Hour’ program and Group B comprised 169 patients seen after the implementation. No significant differences were found in age, sex, treatment phase or complete blood count (CBC). Group B also included patients with severe aplastic anemia due to the high rates of infectious complications in these patients, resulting in a significant difference between the diagnoses of the two groups.

### Group A

Of the 122 patients in this group, the median age was six years (range: 1–15 years); 71 (58 %) were male and 51 (42 %) were female. The diagnosis, treatment phases and CBC values are listed in Table 1.

**Table 1:** Characteristics of febrile neutropenia of patients before (Group A) and after (Group B) the intervention.

Variable	Group A (*n* = 122)	Group B (*n* = 169)	*p*-value
Age - median years (range)	6 (1–15)	5 (1–15)	0.74
Gender - (%)			
Male	71 (58)	105 (62)	0.49
Female	51 (42)	64 (38)	
Diagnosis - n (%)			
ALL	74 (60)	93 (55)	<0.000
Solid Tumor	20 (16.3)	36 (21.3)	
AML	12 (9.8)	16 (9.5)	
CNS tumor	9 (7.3)	2 (1.2)	
HL	6 (5)	0 (0)	
NHL	0 (0)	2 (1.2)	
Others	1 (0.8)	19 (11.2)	
CBC at admission - median (range)			
Hemoglobin (g/dL)	8.6 (3.4–12.4)	8.7 (5.2–13.2)	0.341
Absolute neutrophil count (cells/µL)	219 (0–956)	93 (0–935)	
Platelets (10^3^/µL)	92.1 (1.5–40.1)	55.1 (32–76.4)	
Treatment phase*	21 (28.3)	23 (13.6)	
Induction			
Consolidation	12 (16.2)	17 (10)	0.089
intermediate maintenance	1 (1.3)	2 (1.2)	
intensification	10 (13.5)	22 (13)	
maintenance	7 (9.4)	11 (6.5)	
relapse/refractory	14 (18.9)	15 (9)	
Post-HSCT	6 (8.1)	2 (1.2)	
Palliative	3 (4)	1 (0.6)	

ALL: acute lymphoblastic leukemia; AML: acute myeloid leukemia; HL: Hodgkin lymphoma; NHL: non-Hodgkin lymphoma; CNS tumor: central nervous system tumor; TTA: Time to antibiotic administration; CBC: complete blood count; HSCT: hematopoietic stem cell transplantation; PICU: Pediatric Intensive Care Unit.

⁎ = Treatment phase for ALL.

**Figure 2:**
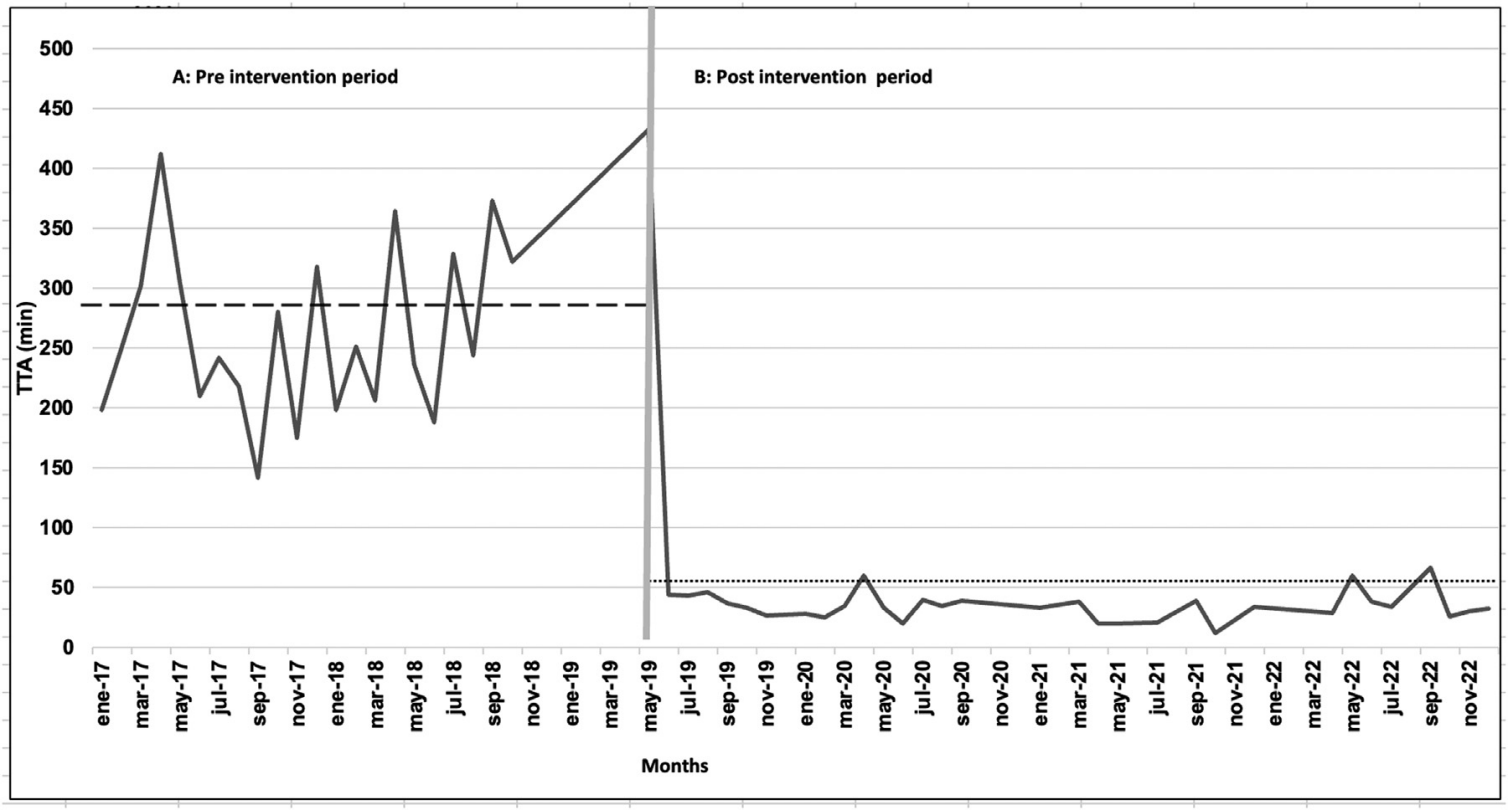
Median time to antibiotic administration of pediatric hematology–oncology patients with febrile neutropenia before (January 2017–March 2019) and after (May 2019–November 2022).

The median TTA was 269.4 min (range: 29–487) (Figure 2), median TTI was 125 min (range: 18–212) and median of TIA was 100 min (range: 10–280). Only seven patients (5.7 %) were administered the first dose of antibiotics within the first 60 min. Imipenem was initially administered to 120 patients (98.3 %), and cefepime was administered to the remaining children. Fifty-three patients (43.4 %) received Vancomycin.

The reasons for delays were: delays in the collection, transportation, and processing of laboratory samples (25 %), difficulty in venous access (20 %), physician delayed making a therapeutic decision (15 %), lack of supplies for antibiotic administration (12 %), and insufficient personnel in the ED (15 %)

In the historical group, culture sampling was not standardized and, often due to the lack of availability of culture media, blood samples for cultures were drawn 12–24 h after the patient's arrival, after one or several doses of antibiotics, and generally only one blood culture was carried out. At least one blood and urine culture was taken from all patients in Group A. Only ten positive cultures were reported, four urine cultures, and two bronchial secretion cultures. A total of 12 documented infections (9.8 %) were reported in this group of patients with some type of antibiotic resistance being identified in three cultures. The microorganisms identified in this group of patients are listed in Table 2.

**Table 2:** Clinical evolution of febrile neutropenia oh patients before (Group A) and after (Group B) the intervention.

Variable	Group A (*n* = 122)	Group B (*n* = 169)	*p*-value
Antibiotic <60 min60 minutes	7 (5.7)	143 (84.6)	<0.000
TTA - minutes (range) Clinical evolution	269.4 (29–487)	50.54 (2–347)	<0.000
Sepsis	14 (11.4)	5 (3)	0.02
PICU	8 (6.6)	4 (2.3)	0.03
Mortality	5 (4.1)	3 (1.7)	0.58
Days hospitalized - mean (range) Antibiotic - n (%)	9.4 (2–62)	7.31 (1–44)	0.017
Imipenem	120 (98.3)	169 (100)	
Vancomycin	53 (43.4)	41 (24.3)	
Other	10 (8.1)	9 (5.3)	
Microorganisms identified - n (%)			
Total	10 (8)	31 (18)	(0.01)
E. coli	1 (0.8)	10 (5.9)*	
Staphylococcus epidermidis	1 (0.8)	6 (3.6)*	
Pseudomonas aeruginosa	1 (0.8)	4 (2.4)	
Klebsiella pneumoniae	3 (2.4)*	3 (1.8)*	
Enterobacter cloacae	0 (0)	3 (1.8)	
Proteus mirabilis	0 (0)	2 (1.2)*	
Staphylococcus aureus	0 (0)	2 (1.2)	
Enterococcus faecalis	0 (0)	2 (1.2)	
Streptococcus mitis	0 (0)	2 (1.2)	
Staphylococcus hominis	0 (0)	1 (0.6)	
Candida albicans	0 (0)	1 (0.6)	
Acinetobacter baumannii	2 (1.6)*	0 (0)	
Corynebacterium spp.	1 (0.8)	0 (0)	

PICU: Pediatric Intensive Care Unit.

⁎ = Resistant.

The mean hospital stay was 9.4 days (range: 2–62 days). A readmission rate of 4 % was reported within the first 30 days for this group of patients.

Fourteen patients (11.5 %) developed sepsis. Eight patients were admitted to the pediatric intensive care unit (PICU) and required critical interventions (6.6 %) with a mean stay of 7.2 days. The remaining patients with sepsis were managed in the pediatric ED. The mortality rate in Group A was 4.1 %, and in all patients the cause of death was septic shock.

### Group B

Of the 169 patients evaluated in the post-intervention period, the median age was five years (range: 1–15 years), 105 (62 %) were male and 64 (38 %) were females. The diagnosis, treatment phases and CBC values are shown in Table 1.

The median TTA was 50.54 min (range: 2–347) (Figure 2) achieving the administration of the first dose of antibiotics within the first 60 min in 84.6 % of patients. All patients received imipenem as the first antibiotic. The addition of a second antibiotic (vancomycin) was necessary for 25 % of the patients. Amphotericin B was added in three patients because of the clinical suspicion of mycosis.

The causes for a delay in the administration of antibiotics were difficulty in venous access (76.9 %) and insufficient personnel in the ED (23 %). The strategy of standardizing culture sampling (two blood cultures and urinary cultures were obtained for all patients) increased the number of infectious agents isolated, allowing for the adjustment of treatment regimens according to the microorganism and antibiotic sensitivity: 31 infections were documented (18 %); 19 were positive blood cultures, 11 were positive urine cultures, and one positive for both cultures.

Five patients (3 %) developed sepsis. Critical care interventions and admission to the PICU were required by four patients (2.3 %). A mortality rate of 1.7 % (3 patients), all due to septic shock, was reported.

### Comparison of clinical indicators

The clinical impact of intervention was evidenced by a reduction in sepsis (11.5 % versus 3 %: *p*-value = 0.02) and proportion of patients who required critical interventions due to clinical deterioration and admissions to the PICU (6.6 % versus. 2.3 %: *p*-value = 0.03). In addition, it was possible to reduce the length of hospital stay (9.4 versus 7.31 days: *p*-value = 0.017). Although the difference was not statistically significant, the mortality rate reported in the pre-intervention group decreased from 4.1 % to 1.7 % after the intervention (*p*-value = 0.58).

Other factors, such as the chemotherapy phase, that could have influenced the need for critical interventions in patients were analyzed. A higher proportion of critical interventions was documented for the post-hematopoietic stem cell transplant patient group (25 %), patients who were on a second-line regimen (8.3 %), and patients in remission induction (4.5 %). However, these differences did not reach statistical significance (*p*-value = 0.065).

Additionally, the underlying diagnoses of the patients were examined; patients with AML had a higher proportion (11 %) of deterioration and requirement for critical interventions, although this did not reach statistical significance (*p*-value = 0.7)

## Discussion

In LMICs, like Mexico, infections are the main cause of mortality among patients treated for cancer, with a mortality rate ten times higher than in HIC.^26^ Thus, the improvement in TTA is crucial as it has demonstrated high efficiency in reducing complications such as sepsis, and the number of patients admitted to intensive care due to complications.^20^ However, management and support guidelines are mostly developed in HICs, where hospital capacities are very different.

There are few examples of projects implemented in LMIC. In Davao City in the Philippines, a resource-specific algorithm was developed to treat PHOP and adherence was measured; the authors concluded that the intervention made it possible to optimize resources and improve quality of care of pediatric cancer patients.^27^ The Hospital General Tijuana, Mexico has been carrying out a similar program since 2014 that has demonstrated that a multimodal intervention was associated with a significant decrease in TTA.^19^

A systematic review that described different interventions to reduce TTA, identified as core elements the distribution of FN-warning cards to patients, skills training, education of staff and educational updates or feedback, implemented guidelines, algorithms, and checklists for FN treatment.^28^

The ‘Golden hour’ intervention at the current center has proven to be effective in reducing TTA and admission to the PICU consistently over the long-term. Prior to the intervention, only 5.7 % of patients received their first antibiotic dose within 60 min of presenting to the ED, whereas in the post-intervention period, this rate increased significantly to 84.6 %. The median TTA also improved noticeably from 269 min pre-intervention to 50.5 min post-intervention. These findings are consistent with previous studies reporting a median TTA of 40 min.^19^

The ‘Golden Hour’ strategy likely facilitated an increase in the number of infectious agents isolated (9 % versus 18 %) enabling the extension of targeted treatments based on the microorganism and antibiogram. Previously, cultures were not consistently taken, and often due to resource constraints, they were collected 12–24 h after the patient's admission and after the administration of one or more doses of antibiotics.

Furthermore, the present study showed a significant reduction in the rate of clinical deterioration and admission to the PICU after the implementation of the ‘Golden Hour’ intervention. In the pre-intervention group, 6.6 % of patients experienced clinical deterioration requiring PICU admission, compared to only 2.3 % in the post-intervention group. This suggests that the ‘Golden Hour’ intervention not only reduced TTA, but also had a positive impact on preventing clinical deterioration and the need for more intensive interventions. The decrease in the length of hospitalization in centers with limited resources is also a notable point of the program.

Although not statistically significant, the current study also observed a decrease in the mortality rate from 4.1 % in the pre-intervention group to 1.7 % in the post-intervention group. While this decrease did not reach statistical significance, it may be clinically meaningful, as even a small reduction in mortality can have a significant impact on patient outcomes and overall survival rates.

The MAS collaborative team has provided invaluable guidance and support throughout the implementation and sustainability of the project. Their expertise and assistance have been instrumental in enhancing the quality of care delivered to patients. There are numerous examples of partnerships between medical institutions in HICs and LMICs. Notably, the My Child Matters program of the Sanofi Espoir Foundation has successfully funded 55 pediatric cancer projects in LMICs over a span of ten years. Additionally, the International Outreach Program of St. Jude Childrenʼs Research Hospital has had a significant role in this domain. Both projects emphasize key elements for successful interventions, which include ensuring adequate financing, establishing strong and sustained local leadership, providing nursing and medical training, offering constant mentoring and support, and receiving capacity building and government support.^29^, ^30^

In contrast to adult patients, where the MASCC scale is widely used to predict clinical outcomes in patients with fever and neutropenia, employing a scale with sufficient sensitivity and specificity in pediatric patients poses a challenge. While six risk stratification scales for FN in pediatric patients are available in the medical literature only two (Rondinelli et al. and Santolaya et al.) were created and validated in Latin-America. There is not to this day a validate risk stratification scale in a Hispanic-Mexican population. In our center, a study of stratification scales showed that the Rackoff scale had the best performance, for this reason it will be the stratification scale we will use in the future. However, the validation of these scales was carried out starting in 2021 at our center, which is why it was not included in the current study.^31^

This study has some limitations. Firstly, it is a single-center study, which may limit the generalizability of the findings to other settings. Secondly, the study design is ambispective, which may introduce biases and confounders. Due to the nature of the study, the distribution of the groups is heterogeneous, with some factors such as chemotherapy phase and underlying patient diagnosis potentially affecting the results, although they did not show statistically significant differences. Nevertheless, the strength of this study lies in the long-term evaluation of the intervention and its impact on clinical outcomes, which adds valuable evidence to the literature on improving care for children with FN in LMICs.

## Conclusions

In conclusion, this study demonstrates that the ‘Golden Hour’ intervention is effective in reducing TTA and improving clinical outcomes in children with FN in a hospital in Mexico. The sustainability of the intervention over a three-year period in a LMIC setting highlights its potential for implementation in similar settings. Further research is needed to validate these findings and explore the impact of the intervention in other healthcare settings and populations.

## Declarations

### Funding

This research was funded by México in alliance with St. Jude collaborative project and the Department of Hematology of the Universidad Autónoma de Nuevo León, Monterrey, México (S.0595 Rio Arronte).

### Ethics approval

All procedures performed in this study involving human participants were in accordance with the ethical standards of the institutional and national research committee and with the 1964 Helsinki Declaration and its later amendments or comparable ethical standards. The study was approved by local institutional review board (IRB) of Medicine Faculty of the Autonomous University of Nuevo León (UANL).

### Consent to participate

Informed consent was obtained from legal guardians of the participants and informed assent was obtained from all individual participants included in the study.

## CRediT authorship contribution statement

**Julia Esther Colunga-Pedraza:** Conceptualization, Data curation, Writing – review & editing. **Ingrid Gabriela Lopez-Reyna:** Formal analysis, Data curation, Writing – review & editing. **Denisse Natalie Vaquera-Aparicio:** Conceptualization, Data curation, Writing – original draft. **Samantha Paulina Peña-Lozano:** Data curation, Writing – review & editing. **Jafet Arrieta:** Conceptualization, Data curation, Writing – review & editing. **Lucía Elizabeth Hernández-Torres:** Conceptualization, Data curation, Writing – review & editing. **Perla Rocío Colunga-Pedraza:** Conceptualization, Data curation. **Mónica Regalado:** Data curation, Writing – original draft. **Yajaira Valentine Jiménez-Antolinez:** Conceptualization, Data curation, Writing – original draft. **Fernando García-Rodríguez:** Data curation. **Oscar González-Llano:** Conceptualization, Data curation, Writing – original draft, Writing – review & editing.

## Conflicts of interest

None.
